# Apocynin, a Selective NADPH Oxidase (Nox2) Inhibitor, Ameliorates Behavioural and Learning Deficits in the Fragile X Syndrome Mouse Model

**DOI:** 10.3390/biomedicines12122887

**Published:** 2024-12-18

**Authors:** Yolanda de Diego-Otero, Rajaa El Bekay, Francisco García-Guirado, Lourdes Sánchez-Salido, Rosa María Giráldez-Pérez

**Affiliations:** 1 Cellular Biology, Physiology and Immunology Department, University of Córdoba, 14014 Córdoba, Spain; rgiraldez@uco.es; 2 Research Laboratory, Instituto de Investigación Biomédica de Málaga y Plataforma en Nanomedicina-IBIMA Plataforma BIO-NAND, Hospital Civil, 29009 Malaga, Spain; 3 Endocrinology and Nutrition Clinic Unit, Regional University Hospital of Málaga, 29009 Málaga, Spain; 4 CIBER of Physiopathology of Obesity and Nutrition (CIBERobn), Institute of Health Carlos III (ISCIII), 28029 Madrid, Spain

**Keywords:** fragile X syndrome, Apocynin, NADPH-oxidase, hyperactivity, behaviour, learning

## Abstract

**Background/Objectives:** Fragile X Syndrome (FXS) is associated with intellectual disability, hyperactivity, social anxiety and signs of autism. Hyperactivation of NADPH oxidase has been previously described in the brain of the male *Fmr1*-KO mouse. This work aims to demonstrate the efficacy of Apocynin, a specific NADPH oxidase inhibitor, in treating Fragile X mouse hallmarks. **Methods:** Free radicals, lipid and protein oxidation markers and behavioural and learning paradigms were measured after chronic treatment with orally administered vehicle, 10 mg/kg/day or 30 mg/kg/day of Apocynin. **Results**: The results revealed a reduction in testis weight, an increase in peritoneal fat, and no variation in body weight after chronic treatment. Furthermore, a reduction in hyperactivity was detected in Apocynin-treated male Fmr1-KO mice. Additionally, the higher dose of 30 mg/kg/day also improves behaviour and learning in the male Fmr1-KO mice, normalising free radical production and oxidative parameters. Moreover, a reduction in phospho-EKR1 and P47-Phox protein signals was observed in specific brain areas. **Conclusions**: Thus, chronic treatment with Apocynin could lead to a new therapeutic option for the Fragile X Syndrome.

## 1. Introduction

Fragile X Syndrome (FXS) is characterised by intellectual and emotional disabilities, including learning difficulties, anxiety, seizures, hyperactivity, stereotypic behaviours, autism-like symptoms, and heightened sensory sensitivity [1]. Additionally, patients exhibit phenotypic manifestations such as macro-orchidism, connective tissue dysplasia and elongated faces with low-set everted ears. FXS is the most common currently known form of inherited intellectual disability worldwide [2]. It is a genetic disorder caused by the loss of the X-linked *Fmr1* gene product, named fragile X mental retardation protein (FMRP), a mRNA binding protein involved in translational regulation [3,4]. The instability of the CGG repeats in the fragile X mental retardation 1 (*Fmr1*) gene is the most frequent mutation, leading to the absence of FMRP [5]. The prevalence of FXS has been estimated to be 1:4000–6000 in males and 1:6000–11,000 in females [1,6].

The *Fmr1*-knockout (Fmr1-KO) mouse model has been widely utilised in research as a well-established and validated animal model for studying FXS. These mice lack the production of the FMRP protein [7], leading to characteristics such as macro-orchidism, atypical dendritic spine morphology with disrupted synaptic plasticity, hyperactivity, learning deficits, heightened seizure susceptibility, exaggerated responses to auditory stimuli and abnormal anxiety behaviours [8]. Previous studies using the FXS mouse model have demonstrated an increase in reactive oxygen (ROS) and nitrogen (RNS) species generation, as well as nicotinamide adenine dinucleotide phosphate oxidase (NADPH oxidase) activation and a moderate increase in oxidative stress in the brain [9,10,11].

Apocynin, also known as 4-hydroxy-3-methoxyacetophenone or acetovanillone, is present in the extract from the root of *Picrorhiza kurroa*, a perennial herb that is found in the Nepalese Himalayas, identified during activity-guided isolation of immunomodulatory elements from its roots [12]. The anti-inflammatory effects of Apocynin result from its ability to selectively prevent the formation of free radicals [13]. It prevents the translocation of the p47phox subunit to the membrane of different cells, avoiding NADPH complex formation [14,15]. The Mouse model in which the p47 subunit is silenced displays resistance to the induction of amyotrophic lateral sclerosis [16]. In addition, Apocynin was observed to reduce the behaviour and learning deficits in a Parkinson’s disease mouse model [17].

Apocynin functions as a specific inhibitor of NADPH oxidase activity, thereby reducing the production of reactive oxygen species (ROS). The superoxide anion (O_2_•^−^) generated by NADPH oxidase is a key contributor to the development of brain injury. By inhibiting the activation of NADPH oxidase, Apocynin mitigates brain damage in models of experimental ischemic stroke [18,19]. A monkey model of Parkinson’s disease showed improved clinical and pathological features after treatment with Apocynin [20], as well as neuroprotective effects through the attenuation of oxidative damage and neuroinflammatory responses [21]. Moreover, Apocynin, when used in the treatment of Type 4 cardiorenal syndrome (CRS), has been shown to reduce oxidative stress in the kidneys and heart [22]. Additionally, Apocynin derivatives have anti-tumoural effects in the treatment of breast cancer [23].

This work aims to assess the use of Apocynin in an experimental treatment for FXS. We analysed free radical production, lipid and protein oxidation measurements, and changes in NADPH oxidase-mediated intracellular signalling. Additionally, we carried out behavioural studies to determine the effectiveness of Apocynin treatment in normalising the clinical and behavioural phenotype of the Fmr1-KO mouse.

## 2. Materials and Methods

### 2.1. Experimental Animal Model

All experiments utilised the *Fmr1*-knockout FVB-129 mouse strain, generously provided by B. Oostra (Erasmus University Rotterdam, Rotterdam The Netherlands) [7]. The mice were maintained as a colony in the Experimental Animal Facility of the University of Malaga (Málaga, Spain). All procedures adhered to the guidelines established by the University of Malaga Animal Welfare Committee and conformed to the European Communities Council Directive (2010/63/EU, 90/219/EEC, Regulation (EC) No. 1946/2003) concerning the care and use of experimental animals [24]. The animal studies carried out in this work were approved by the Animal Care and Use Committee at the Regional University Hospital of Malaga (12-02-2010). The mice were kept in a controlled environment with regulated temperature and humidity, maintained on a 12 h light/dark cycle, and provided with unrestricted access to standard food and water. Four-month-old male wild-type (WT) control and *Fmr1*-KO mice were used for these experiments. The littermates were produced by crossing heterozygous (wild-type/Fmr1-knockout) females with hemizygous (*Fmr1*-knockout) males and were randomly assigned to various experimental groups comprising 4–12 animals per group. Only male mice were used in the study, contrary to what occurs in other mouse models and despite hormonal changes, most notable findings and differences occur in males rather than females in this mouse model [25].

### 2.2. Apocynin Treatment

Animals were first exposed to a battery of behavioural tests before entering the pre-clinical trial. Then, the mouse group received approximately 10  mg/kg/day or 30 mg/kg/day of Apocynin (Sigma-Aldrich, Buchs, Switzerland) or vehicle as control (each group *n* = 10–12). The mice were treated orally for 30 days in drinking water (see B graphical abstract). Behavioural tests were repeated at the end of the protocol.

### 2.3. Behavioural Analysis

The animals were kept in the test room for 30 min prior to starting the behavioural experiments. All the specific details regarding behavioural procedures are in the Supplementary Material and Methods. Open-field procedures to assess hyperactivity were similar to those described previously [24,26]. The object recognition test to assess cognition is currently one of the most widely used behavioural tests for mice [27]. The elevated plus-maze paradigm to assess anxiety response was based on that designed and validated by Lister [28]. The shuttle box test assessed fear-conditioning hippocampal/amygdala learning [29].

### 2.4. Brain Dissection

Mice were euthanised via cervical dislocation, after which their brains were rapidly extracted and dissected to isolate specific regions, including the hippocampus, prefrontal cortex, and cerebellum, on a chilled surface. The tissues were promptly frozen using liquid nitrogen and stored at −80 °C for subsequent analyses. For ex vivo experiments, the brains were sectioned into 100-µm-thick slices and placed on plates containing sterile KR-HEPES buffer for ROS assessments.

### 2.5. Protein Extraction for Measurements of Oxidative Stress

Tissue samples were homogenised in phosphate buffer (pH = 8) with Kimble^®^ Kontes Disposable Pellet Pestles (Sigma-Aldrich) and posterior sonication (Vibracell^TM^ Ultrasonic, Sonics & Materials, Newtown, CT, USA). Cytosol fractions were obtained as described previously, while membrane fractions were produced by centrifugation at 25,000× *g* for 30 min at 4 °C. The amount of protein was calculated spectrophotometrically according to the Bradford method [30].

### 2.6. Intracellular H_2_O_2_ Production

ROS production was assessed using 2,7-dichlorohydrofluorescein diacetate (DCFDA) as a marker for intracellular H_2_O_2_ levels. In brief, macrophages at a density of 2 × 10^6^ cells/mL were incubated at 37 °C in the dark with 2.5 µM DCFDA dissolved in ethanol. Following incubation, the cells were washed twice with KR-HEPES buffer, and fluorescence intensity was recorded at various time points using a fluorimeter with an excitation wavelength of 503 nm and an emission wavelength of 529 nm, utilising a Clarity Microplate Reader (BioTek Instruments, Inc., Winooski, VT, USA). Furthermore, intracellular ROS production was quantified in brain slices after 24 h of incubation with Apocynin at 37 °C in 5% CO_2_. Protein concentration in cell fractions was determined using the Bradford method [30], and all fluorescence values were normalised and expressed as a percentage relative to control wells.

### 2.7. Oxidative Parameters

All the specific details about procedures are in Supplementary Material and Methods. Lipid peroxidation was determined in brain membrane fractions by the measurement of Thiobarbituric Acid-Reactive Substances (TBARS) as described previously [31]. Protein oxidation was determined by measuring protein carbonyl compounds, the most used marker of protein oxidation, and a slight modification of the previously described methods was used in this experiment [32,33].

### 2.8. Western Blot Analysis

The p47phox protein, an NADPH-oxidase subunit involved in the cell-free radical production (see A graphical abstract), and also the activation of ERK1/2, an intracellular signalling protein activated by ROS, were studied by Western blot analyses and was performed using brain extracts from 4-month-old male mice. Two main structures, the hippocampus and cerebellum, were dissected. The samples were homogenised using a motorised pellet pestle in 1 mL of homogenisation buffer containing 20 mM HEPES and 100 mM KCl at pH 7.0, supplemented with a protease inhibitor cocktail (SigmaFast, cat# 8830, Sigma, Saint Louis, MO, USA). Protein concentration was determined for each sample using the Bradford method. Tissue sample aliquots equivalent to 40 µg of cytosolic protein were mixed with an equal volume of sample buffer (composed of 2% SDS, 5% mercaptoethanol, bromophenol blue, and 20% glycerol), heated to 100 °C for 5 min, and then loaded onto 12% polyacrylamide gels. The separated proteins were transferred to a PVDF membrane, blocked for 1 h at 37 °C in a blocking solution containing 3% BSA, 0.05% Tween-20, and PBS (pH 7.4), and incubated overnight at 4 °C with primary antibodies diluted in the blocking solution. The rabbit polyclonal anti-phospho-specific ERK (cat# sc-7976-R, Santa Cruz Biotechnology, Inc. Santa Cruz, CA, USA) and unphosphorylated ERK1/2 (cat# sc-93, Santa Cruz Biotechnology, Inc., Santa Cruz, CA, USA) and the goat polyclonal anti-p47phox (cat# sc-7660, Santa Cruz Biotechnology, Inc. Santa Cruz, CA, USA). The β-actin (cat# A5060, Sigma-Aldrich, St. Louise, MO, USA). The membranes were washed three times for 10 min each with PBS containing 0.05% Tween-20, followed by a 1-h incubation at room temperature with a 1:5000 dilution of goat anti-rabbit IgG-HRP (cat# sc-2004, Santa Cruz Biotechnology, Inc., Santa Cruz, CA, USA) in PBS supplemented with 3% BSA. Afterwards, the membranes were rinsed three additional times for 10 min each and processed for detection using an enhanced chemiluminescence kit (cat# 170-5070, Bio-Rad, Hercules, CA, USA). Signal visualisation was performed using a digital luminescent image analyser (Bio-Rad, Hercules, CA, USA).

## 3. Results

### 3.1. Chronic Apocynin Treatment Did Not Affect Total Body Weight

The possible effects of the amount of Apocynin, a p47-phox subunit inhibitor, on the welfare of mice was measured based on the weights of the animals over 120 days (Figure S1A Supplementary). Weight is displayed as a minimum variation, with no significant difference between WT control mice in comparison to Fmr1-KO mice. In parallel, another experiment was conducted to observe the effect of treatment with a vehicle and two different doses of Apocynin on weight. Vehicle or 10 mg/kg/day Apocynin or 30 mg/kg/day Apocynin was administered orally in drinking water to Fmr1-KO mice and WT mice for 30 days, during which the mouse weight was monitored. No differences in weight were noted (Figure S1B Supplementary). Figure S1C Supplementary shows the weight of testes, and Figure S1D Supplementary shows the peritoneal fat of mice after chronic treatment with Apocynin. Despite macroorchidism not being normalised after chronic treatment, a dose-dependent tendency for weight reduction was observed in the Fmr1-KO. Moreover, a statistically significant reduction of the peritoneal fat weight was observed in the vehicle-Fmr1-KO group compared to the WT-control group. Additionally, a normalisation of the peritoneal fat weight was observed in the Fmr1-KO mice treated with both doses of Apocynin compared to the vehicle Fmr1-KO-group (Figure S1D Supplementary). Furthermore, the treatment did not affect the weight of the other body tissues in the treated groups.

### 3.2. Apocynin Normalized Free Radical Production in the Fmr1-KO Mouse Model

**Figure 1:**
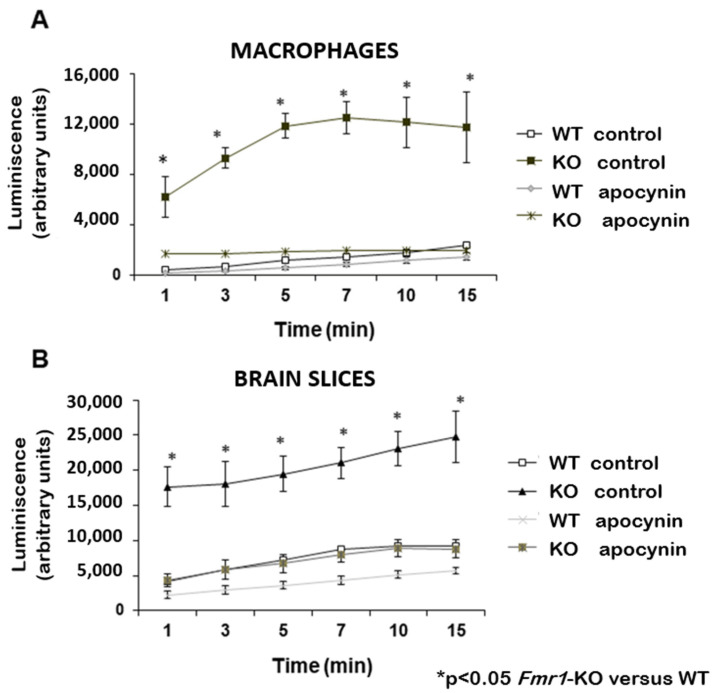
Measurement of the effect of Apocynin on ROS production in macrophages (**A**) and brain slices (**B**). A reduction of ROS production was observed after chronic treatment with 30 mg/kg/day of Apocynin. The data show the mean luminescence (arbitrary units) measured in three mice per group for each time point ± SEM (* *p* < 0.05 Fmr1-KO versus WT).

Using the lucigenin chemiluminescence technique to measure free radicals in mouse peritoneal macrophages, Figure 1A shows a significant increase in superoxide anion production (O_2_•^−^) in the Fmr1-KO compared with the WT-controls. This increase was completely blocked by chronic oral treatment in the drinking water with 30 mg/kg/day.

Apocynin for 30 days (Figure 1A). Using the same technique to measure superoxide production in brain slices, a significant increase of 6 to 8 times was detected in the production of superoxide (O_2_•^−^) in Fmr1-KO mice compared with the WT-control group. The production of free radicals in brain explants of Fmr1-KO mice was normalised in response to chronic treatment with 30 mg/kg/day Apocynin for 30 days (Figure 1B).

### 3.3. Apocynin Reverses Oxidative Stress in the Fmr1-KO Mouse Model

**Figure 2:**
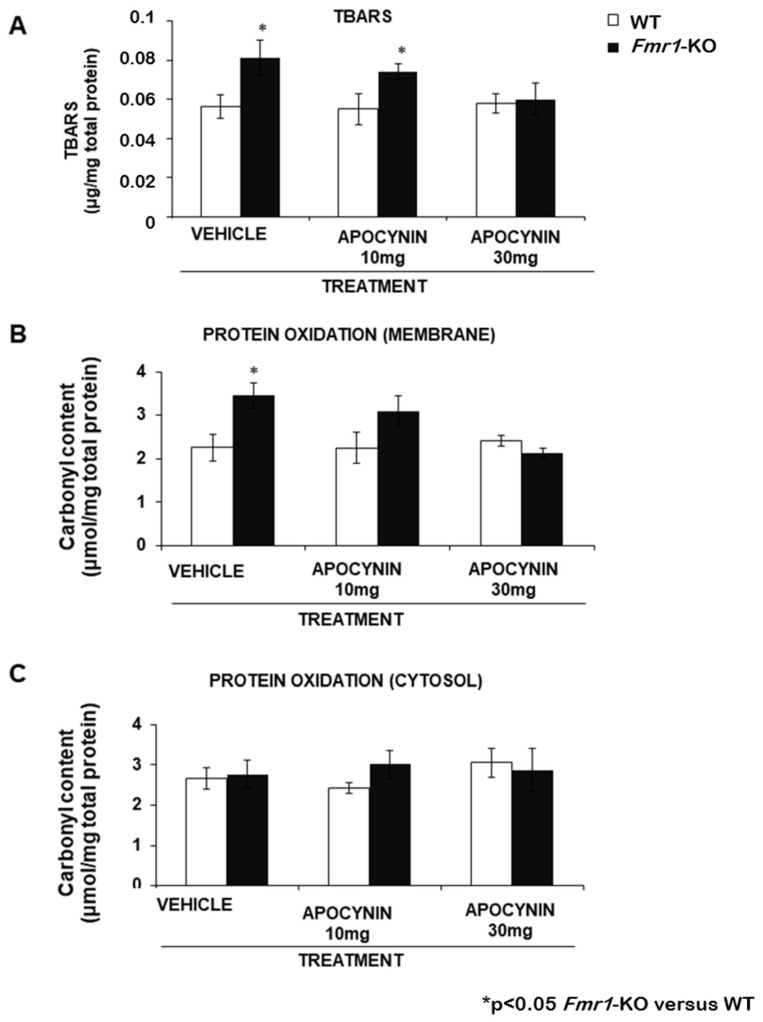
Apocynin treatment reduced oxidation levels in lipids (**A**) and proteins (**B**,**C**). Apocynin reduced brain TBARS levels when the Fmr1-KO group was compared to the WT-control group. Determination of the membrane lipid peroxidation fraction from the brain by quantification of the spectrophotometry conducted for TBARS in WT and Fmr1-KO mice after chronic treatment with vehicle and Apocynin (10 mg/kg/day and 30 mg/kg/day). Carbonyl content of protein from the membrane and cytosolic fractions isolated from WT-control and Fmr1-KO brains after chronic treatment with vehicle and Apocynin (10 mg/kg/day and 30 mg/kg/day). Data are expressed as mean values ± S.E.M. Statistical significance was determined using the unpaired *t*-test, and * *p* values < 0.05 were considered significant when the Fmr1-KO group was compared to the WT-control group.

A Lipid peroxidation index was assessed in the plasma membrane fraction of brain samples from Fmr1-KO and WT-control mice chronically treated with either vehicle or the test compound administered orally, dissolved in the drinking water. Thiobarbituric acid-reactive substances (TBARS) levels were measured in brain membranes, and results revealed a significant TBARS increase in the Fmr1-KO group compared to the WT-control group under vehicle conditions and following chronic treatment with 10 mg/kg/day Apocynin. However, chronic administration of a higher dose of 30 mg/kg/day of Apocynin effectively stabilised TBARS levels when the Fmr1-KO group was compared to the WT-control group (Figure 2A).

The effectiveness of Apocynin treatment was additionally analysed by measuring a well-known marker of protein oxidation. The carbonyl content of protein was measured in brain membrane protein, and results revealed a significant increase in this marker of protein oxidation in the vehicle Fmr1-KO groups, which was normalised with chronic 30 mg/kg/day Apocynin treatment (Figure 2B). The carbonyl content in the cytosol in the vehicle Fmr1-KO groups did not show a significant increase, and this situation was maintained regardless of the treatment (Figure 2C).

### 3.4. Chronic Apocynin Treatment Normalised Behavioural Hallmarks in the Fmr1-KO Mouse Model

**Figure 3:**
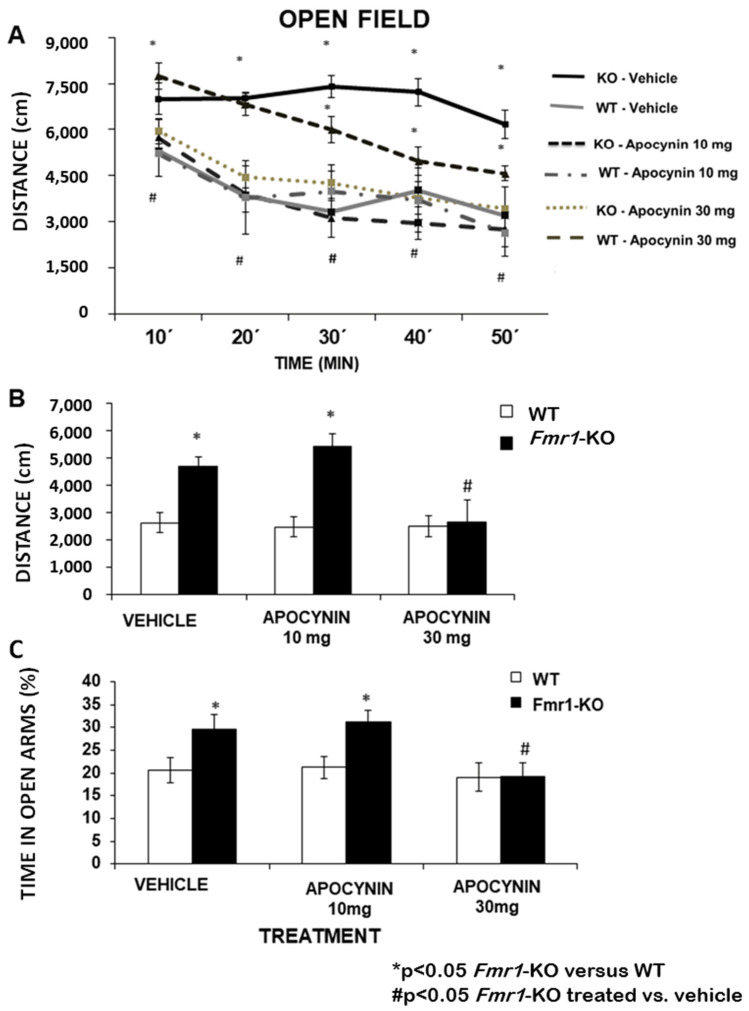
The open field maze was used to assess hyperlocomotion in a novelty condition. The mice’s activity in the maze was tracked with a digital video camera coupled with Smart 3.0—Video Tracking System (SMART30) Harvard Bioscience (https://www.panlab.com/es/productos/smart-video-tracking-software-panlab, accessed on 14 December 2024). (**A**) Distance runs in each 10-min interval during the first day (novelty) of the open field maze, *Fmr1*-KO mice treated with vehicle (*n* = 10) and 10 mg/kg/day Apocynin (*n* = 8) covered more distance than WT mice that received the same treatment (*n* = 8 and *n* = 7, respectively). However, the *Fmr1*-KO mice (*n* = 5) treated with 30 mg/kg/day Apocynin displayed no significant difference between WT (*n* = 7) mice with the same treatment. (**B**) Locomotion activity during the 15-min test in the open-field maze in the familiarity environment of Fmr1-KO and WT-control male mice chronically treated with vehicle and 10 mg/kg/day and 30 mg/kg/day Apocynin. Data are presented as mean values of activity ± S.E.M. # *p* < 0.05 treated-*Fmr1*-KO versus vehicle-*Fmr1*-KO; * *p* < 0.05 Fmr1-KO versus WT-control. Values of *p* < 0.05 were considered significant. (**C**) Correction of anxiety with chronic Apocynin treatment. The effects of two concentrations of Apocynin (10 mg/kg/day and 30 mg/kg/day) on the behaviour of *Fmr1*-KO male mice in the elevated-plus-maze. Vehicle and 10 mg/kg/day Apocynin did not affect anxiety response (average time spent in the open arms was elevated in *Fmr1*-KO). After chronic daily treatment with 30 mg/kg/day Apocynin, the anxiety response was normalised (significantly affected the amount of time mice spent in the open arms of the maze). The elevated plus maze assesses time spent in the open arm. Data are presented as the mean percentage of time in the open arms ± S.E.M (*n* = 5–10). # *p* < 0.05 treated-KO versus vehicle-KO; * *p* < 0.05 *Fmr1*-KO versus WT-control. Values of *p* < 0.05 were considered significant.

To characterise whether the abnormal behaviour, such as hyperactivity and anxiety responses observed in the FMR1-KO mouse model, improved with the apocynin treatment, we employed the Open Field paradigm to assess hyperlocomotion in novelty and familiarity conditions and also to examine the habituation profile. After chronic treatment with vehicle and 10 mg/kg/day Apocynin, the Fmr1-KO group exhibited a notable increase in activity compared to the WT-control group under both conditions. However, normalisation of hyperlocomotor activity was observed in the Fmr1-KO group following chronic administration of 30 mg/kg/day Apocynin (Figure 3A), with no significant difference detected between Fmr1-KO and WT-control mice.

A significant increase was observed in the distance travelled by the vehicle Fmr1-KO group during the total 50-min test on the first day in novelty conditions (Figure 3A) and during the 15 min on day 2 of the test in familiarity conditions (Figure 3B). Then, in novelty and familiarity conditions, the Fmr1-KO group showed normalisation in locomotor activity in response to the high dose of 30 mg/kg/day Apocynin compared with the WT-control.

Anxiety response in the mouse groups was measured using the elevated plus maze, which is based on the natural aversion of mice to open and elevated areas [9]. In this case, we measured the time spent in the open arm to determine the effect of Apocynin on the anxiety response. The percentage of time spent in the open arms (Figure 3C) was increased in Fmr1-KO in comparison to the WT-control mice, under vehicle and 10 mg/kg/day Apocynin treatment, reflecting a reduced anxiety response in the Fragile X mouse model. However, the average time spent in the open arms of the maze was normalised with 30 mg/kg/day Apocynin, indicating a normalisation of the anxiety response.

### 3.5. Chronic Apocynin Treatment Normalised Cognitive Hallmarks in the Fmr1-KO Mouse Model

**Figure 4:**
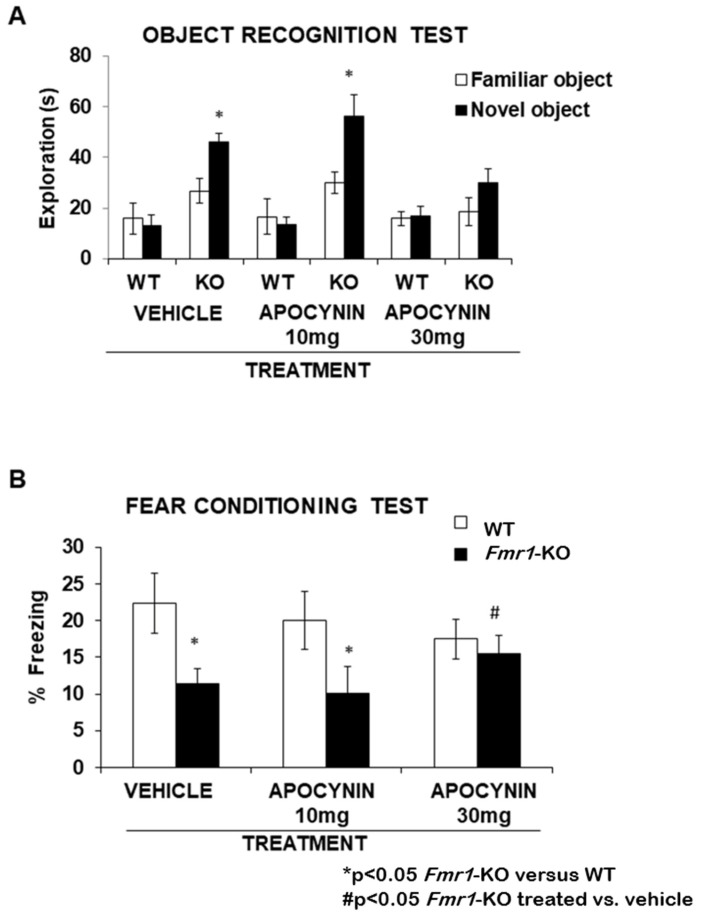
(**A**) The lack of FMRP protein disrupts cued fear conditioning, while chronic treatment with 30 mg/kg/day Apocynin alleviates the learning deficits observed in the *Fmr1*-KO mouse model. Twenty-four hours after a training session, mice underwent testing for cued fear conditioning. *Fmr1*-KO groups treated with vehicle or 10 mg/kg/day Apocynin exhibited significantly lower freezing levels during the test compared to the WT-control group. Nevertheless, chronic administration of 30 mg/kg/day Apocynin restored these learning impairments, highlighting hippocampal and amygdala memory deficits in the Fmr1-KO mice. Data are presented as % freezing means ± S.E.M., with a sample size of 5–10 mice per group. Statistical significance was set at *p* < 0.05, where # *p* < 0.05 indicates differences between treated-KO and vehicle-KO groups; * *p* < 0.05 indicates differences between *Fmr1*-KO and WT-control. (**B**) Learning, encompassing both short-term and long-term memory, was assessed using the object recognition test. High-dose Apocynin treatment reduced the time *Fmr1*-KO mice spent exploring the novel object compared to vehicle-treated *Fmr1*-KO mice (# *p* < 0.05 indicates significant differences between treated-KO and vehicle-KO groups; * *p* < 0.05 for *Fmr1*-KO versus WT-control). Data represent the mean object exploration time ± S.E.M. (*n* = 8–10 mice).

The effect of treatment with Apocynin on short-term and long-term memory was measured using the object recognition test. This test is based on the natural tendency of mice to explore a novel object with respect to a familiar object. Figure 4A shows that the vehicle Fmr1-KO group spent a significantly higher amount of time exploring the new object compared to the WT-control group. The same was observed in the treatment with Apocynin 10 mg/kg/day; a significant increase was observed in the time spent exploring a novel object in the Fmr1-KO group with respect to the WT-control group. However, chronic treatment with the higher dose of Apocynin (30 mg/kg/day) showed an improvement in short-term memory that was associated with a reduction in the time exploring the novel object in the paradigm of the new object recognition test.

Learning deficit was assessed to study the effectiveness of chronic Apocynin treatment on cognition; we analysed fear learning conditioning on the Shuttle-box paradigm. The Fmr1-KO group, compared to the WT-control group, showed no difference in freezing response in contextual experiments performed in the testing session with our experimental conditions. The Fmr1-KO group, compared with the WT-control group, showed a reduced freezing response in cued experiments during the testing session in the vehicle group and Apocynin 10 mg/kg/day group (Figure 4B). However, Fmr1-KO mice chronically treated with 30 mg/kg/day Apocynin normalised the cued freezing response during fear conditioning learning.

### 3.6. Chronic Apocynin Treatment Reduces Activation in Intracellular Oxidative Pathway in the Fmr1-KO Mouse Model

**Figure 5:**
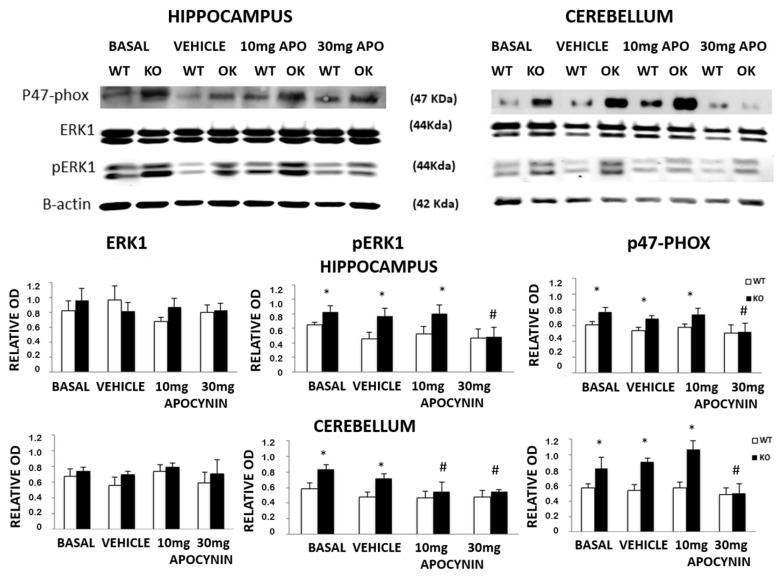
pERK1/2 and p47 were highly phosphorylated in the brain of Fmr1-KO compared with WT-control mice, and no differences were detected in ERK1/2. The animals treated with a high dose of Apocynin showed a significant reduction of the phosphorylated forms compared with vehicle-treated Fmr1-KO (* *p* < 0.05 Fmr1-KO versus WT-control; # *p* < 0.05 Fmr1-KO Apocynin-treated versus vehicle-treated Fmr1-KO). The data in the graph represent the mean ± SEM (*n* = 4). Whole brains from 4-month-old Fmr1-KO and WT-control mice were lysed as described in Materials and Methods. An amount of 45 μg/lane of protein extracts was subjected to SDS-PAGE, therefore transferred to PVDF membrane, and subsequently probed with anti-phospho-ERK1/2, anti-ERK1/2 or anti-p47, and then, accordingly to the protocol, the corresponding bands were detected by luminol-enhanced chemiluminescence. To verify even protein loading, the blots were subsequently stripped and reprobed with antibodies against β-actin. Blots are representative of a set of two experiments that generated similar results.

In order to understand the effects of the Apocynin treatment over P47phox, a protein subunit involved in the activation of NADPH-oxidase to produce free radicals, and also to understand the effects of the activation of ERK1/2, a protein that will be activated by free radical production, Western-blotting studies were performed on proteins extracted from dissected brain areas to explore ERK1/2, pERK1/2 and p47phox proteins normalised by the Beta-actin protein level, during chronic Apocynin treatment of the *Fmr1*-KO and the WT-control mice. The level of the ERK1/2 and pERK1/2 proteins was evaluated using specific antibodies for the normal or phosphorylated form of these enzymes. Figure 5 shows that p47phox and pERK1/2 were increased in two brain areas such as the cerebellum and hippocampus of the Fmr1-knockout group compared to the wildtype-control group, whereas non-phosphorylated ERK1/2 levels were similar to both genotypes. Western blot analysis revealed that phosphorylated forms of these two proteins, pERK1/2 and p47phox, showed a normalisation after 30 mg/kg/day Apocynin chronic treatment, while ERK1/2 levels were not changed in each group of mice after treatment (Figure 5).

## 4. Discussion

The brain of the Fmr1-KO mouse showed a functional hyperactivation of NADPH-oxidase in several areas. Apocynin, a specific inhibitor of NADPH oxidase, has been used for the first time to treat Fmr1-knockout mouse hallmarks, such as oxidative stress markers (free radicals, lipid and protein oxidation) and to improve behavioural and learning paradigms.

Although previous studies have shown that treatment with mGlur5 antagonist or GABA agonist reduces behaviour problems in the Fmr1-KO mice, such as social approach deficits, repetitive self-grooming, high marble burying scores and stereotypical behaviour, implicating several neurotransmitter pathways in the physiopathology of this syndrome [34,35], our study represents another approach to experimental treatment for the Fragile X pathology, working under the hypothesis of brain oxidative stress [9,10,11,24]. The present study shows that oral chronic administration of 30 mg/kg/day Apocynin, a nontoxic herbal compound with anti-inflammatory potential acting as NADPH oxidase assembly inhibitor, improves the behavioural symptoms of the FXS in the Fmr1-KO mouse model. The attenuation of FXS pathogenesis by Apocynin was accompanied by the normalisation of oxidative parameters. Apocynin acts as a selective inhibitor of NADPH oxidase by reducing ROS production, as previously described in human neutrophils [15] and in the brain [36].

The two daily treatment regimens for chronic administration of Apocynin were more effective at the highest dose of 30 mg/kg/day. This dose improves learning, locomotor activity and the anxiety response in the FXS mouse model. The lower dose of Apocynin (10 mg/kg/day) has not improved the typical behaviour of FXS in the different behavioural tests assessed in the study.

To test the tolerance and safety of Apocynin in mice, a dose-response curve over the body weight was performed for 120 days, scaling the dose up to 200 mg/kg/day. In the same way, the effect on body weight of the doses used for chronic treatment (10 mg/kg/day and 30 mg/kg/day) was studied, and no variation was observed. Furthermore, mortality was not affected. Therefore, this finding may indicate that Apocynin is well tolerated by the strain of mice used in these experiments (FVB), as an increased dose of Apocynin did not produce significant changes in weight or mortality in the mice. It has also been demonstrated that in 4-week-old C57BL/6J mice (other strain) given a high-fat diet, Apocynin treatment by oral application (water) does not cause a weight change [37].

Mice without FMRP express macroorchidism [8]. In this study, the abnormally large testes were maintained in Fmr1-KO mice treated with Apocynin, and these FXS phenotypic characteristics were not reversed, although a statistical trend was observed towards a reduction in size with the higher administered dose of 30 mg/kg/day. However, there are results in our previous publication showing that antioxidant chronic treatment with alpha-tocopherol reduced the enlarged testicles in the Fmr1-KO mice [9], and also in the results published where treatment with a selective MNK Inhibitor also reduced macro-orchidism [38].

In reference to hyperactivity, in the present study, Fmr1-KO animals chronically treated with 30 mg/kg/day Apocynin displayed reduced locomotion levels. These results are in agreement with those observed in other hyperlocomotion models [39,40].

Apocynin at the cognitive level has been shown to be useful in improving learning deficits in high oxidative stress models of traumatic brain injury. In these cases, the learning deficits are reverted when high concentrations of Apocynin are employed, but not with lower doses of the inhibitor [41]. The results observed in these experiments are consistent with previous works, as they showed a normalisation of learning in object recognition tests; Fmr1-KO mice treated with a chronic daily dose of 30 mg/kg Apocynin showed a reduction of the analysed parameters. In addition to the results of the object recognition test, an improvement of the cued learning response was also achieved in the fear conditioning test. In this behavioural test, the Fmr1-KO mice treated with 30 mg/kg/day recovered from abnormal cued learning.

Other studies have shown how oxidative stress is involved in neurodegenerative diseases such as AD [42], PD [43] and other pathologies with inflammatory processes [17] and associated with anxiety in different animal models [44,45,46]. Additionally, it has a specific role in the development of anxiety responses in different psychiatric pathologies [47]. In this study, treatment with Apocynin resulted in a reduction of oxidative stress markers, in accordance with previous studies performed on cellular analysis [48]. Apocynin is a well-known experimental drug derived from a plant flavonoid, with an oral toxicity prediction classifying it as a safe compound (Class 6, predicted LD50 of 9000 mg/kg) [49]. It has demonstrated the ability to reduce reactive oxygen species (ROS) production and protect against cellular damage. It has been found to suppress pro-inflammatory responses by inhibiting the activation of nuclear factor kappa B (NF-κB). The anti-inflammatory properties of apocynin are likely linked to its capacity to decrease ROS levels, which is attributed to its antioxidant activity. Additionally, apocynin prevents protein oxidation, a process that can contribute to cell death. It has been extensively used in therapeutic strategies to combat neuronal damage and has been found to regulate anxiety in animal models [50]; our experiments were performed on male mice because they showed the most prominent symptoms of the disorder, females showed mild symptomatology, and several publications have also presented results using only male FMR1-KO mice [51,52]

ERK1/2 kinases and p47 are involved in cellular responses to stress stimuli, including free radical production, oxidative stress, glutamate receptor stimulation, or an increase in intracellular calcium levels, which play a critical role in harmful microglia activation in acute brain injury and neurodegeneration [53,54]. In addition, ERK1/2 protein plays a pivotal role in dendrite growth and has been involved in memory storage and neuronal long-lasting plasticity [55]. The activation of ERK1/2 observed in this study could be a consequence of NADPH oxidase activation in brain areas from Fmr1 knockout mice, resulting in intracellular hyperphosphorylation, and this abnormal signalling was controlled under chronic treatment with Apocynin. Our results are in agreement with previous results performed in a mouse ageing model, indicating that Apocynin reduces activation and translocation of p47 [24].

## 5. Conclusions

In conclusion, the current study suggests that Apocynin could represent a progression in the development of an experimental treatment for Fragile X Syndrome. The present study demonstrates that chronic daily treatment with 30 mg/kg/day Apocynin normalises hyperlocomotion and anxiety response, improves learning and normalises free radical production, reducing intracellular NADPH oxidase signalling and also the normalisation of the oxidation of lipids and proteins in the Fmr1-KO mouse. This new approach may represent a novel option for future therapeutic intervention in Fragile X syndrome.

## Data Availability

All datasets will be shared upon request.

## Supplementary Materials

The following supporting information can be downloaded at https://www.mdpi.com/article/10.3390/biomedicines12122887/s1, 1. Behavioural Analysis. 2. Oxidative parameters. 2.1 Determination of Thiobarbituric Acid-Reactive Substances. 2.2 Protein Oxidation Assays. Figure S1: Measure of Apocynin tolerance in mice. References [26,27,28,29,31,32,33,56,57] are cited in the Supplementary Materials.
