# Supplementary material for: Apocynin, a Selective NADPH Oxidase (Nox2) Inhibitor, Ameliorates Behavioural and Learning Deficits in the Fragile X Syndrome Mouse Model
Source: Biomedicines. 2024 Dec 18;12(12):2887. doi: 10.3390/biomedicines12122887 (PMC11673502; doi:10.3390/biomedicines12122887)
Supplement: Supplementary file 1 [file biomedicines-12-02887-s001.zip › biomedicines-3352708-supplementary.pdf]

# Apocynin, a Selective NADPH Oxidase (Nox2) Inhibitor, Ameliorates Behavioral and Learning Deficits in the Fragile X Syndrome Mouse Model

Yolanda de Diego-Otero[1*#], Rajaa El Bekay[2,3,4#], Francisco Garcia-Guirado[2], Lourdes Sanchez-Salido[2], Rosa María Giráldez-Pérez[1].

1. Cellular Biology, Physiology and Immunology Department. University of Córdoba. 14014. Córboda. Spain.
2. Instituto de Investigación Biomédica de Málaga y Plataforma en Nanomedicina- IBIMA Plataforma BIO-NAND. Research laboratory. Hospital Civil. 29009. Malaga. Spain.
3. Endocrinology and Nutrition Clinic Unit, Regional University Hospital of Málaga, 29009 Málaga. Spain.

rajaa.elbekay@ibima.eu

4. CIBER of Physiopathology of obesity and nutrition (CIBERobn), Institute of Health Carlos III (ISCIII), Spain.

# These authors contributed equally to this work

* Dr. Yolanda de Diego Otero. Cellular Biology, Physiology and Immunology Department. University of Córdoba. 14014. Córboda. Spain.

**1. Behavioral Analysis**

The animals were kept in the test room for 30 min prior to starting the behavioral experiments.

**Open-field** procedures were similar to those described previously [1]. Briefly, each experimental animal was placed in a softly illuminated observation cage (110 x 50 x 50 cm). Activity in the open-field maze was tracked using a digital video camera coupled to SMART software (PANLAB, Barcelona, Spain). The maze was virtually divided into two areas, center and perimeter; we recorded the percentage of time spent in each area during 10-min intervals over a period of 50 minutes as novelty, as well as the number of crosses between areas and the total distance travelled. The percentage of time spent in each area during 15 minutes, the number of crosses between areas and the total distance travelled were recorded during the second day of the test.

**Object recognition test**. The object recognition test is currently one of the most widely used behavioral tests for mice [2]. Briefly, a mouse is presented with two similar objects during the first session (10 minutes), and then one of the two similar objects is replaced with a new object in a second session (10 minutes). This text measures the amount of exploration time of the new object, providing an index of recognition memory. As in the open-field test, activity was tracked using a digital video camera coupled to SMART software (PANLAB, Barcelona, Spain).

The elevated **plus-maze** paradigm was based on that designed and validated by Lister [3], and this test provides a measure of the anxiety status of mice. The anxiety response was characterized by exposure to the plus maze behavioral paradigm in the novelty condition. Each mouse was placed in the central square (5 x 5 cm) facing an open arm and allowed to explore the maze for 5 min. Behavior was tracked in the maze using a digital video camera coupled to SMART software (PANLAB, Barcelona, Spain). Different variables were analyzed, such as the time spent in each zone of the maze (open arms, closed arms and central square); open-arm entries and

closed-arm entries (in both cases, the absolute number as a percentage of total arm entries). The maze was thoroughly cleaned with a dry cloth between sessions.

**Shuttle box**. This test assessed hippocampal/amygdala learning (fear-conditioning). On the first day, the mice were placed individually in a clear methacrylate chamber (PANLAB, Barcelona, Spain) for fear-conditioning training [4]. A 30-s tone of 80-dB [conditioned stimulus (CS)] was activated after a 2-min acclimation period in the chamber. The mice received a 2-s foot shock [unconditioned stimulus (US)] of 0.2 mA intensity during the last 2 s of CS. The mice remained in the chamber for 30 s after the foot shock. This cycle was repeated 3 times, and 24 hours later, the mice returned to the chamber for a contextual test (5 minutes) in the absence of the tone and foot shock. After 90 minutes, the mice were exposed to a different chamber (a different shape but the same area, with a black plastic floor and black and white walls, as well as with a lemon scent to distract the mouse) for a cued test interval (5 minutes), in which the CS tone was offered during the final 3 min. The freezing behavior was measured during conditioning, training and testing. The behavior of the mice was classified as frozen whenever a complete absence of motion was evident. The percentage of frozen time was calculated for each mouse.

## *2. Oxidative parameters*

### 2.1 Determination of Thiobarbituric Acid-Reactive Substances

Malondialdehyde (MDA) is the most abundant lipid oxidant product, and it is used as an index of oxidative stress status. We determined the amount of MDA in the membrane extract by quantifying thiobarbituric acid-reactive substances (TBARs). Lipid peroxidation was determined in brain membrane fractions, as described previously [5]. Briefly, this method is based on the production of TBARS from the reaction between 20% thiobarbituric acid and membrane extract at 95ºC for a duration of 10 minutes. The product of this reaction was measured based on the spectrophotometric absorbance at 532 nm (VERSAmax Molecular Devices Sunnyvale, CA, USA). The TBARS measurements were calculated with a standard curve using serial dilutions of malonaldehyde-bis-diethylacetal (MDA) to 1 mM (Sigma Chemical Co.). The final values were expressed as nmol of TBARs per mg of protein determined using the Bradford method.

### 2.2 Protein Oxidation Assays

The formation of carbonyl compounds is actually the most used marker of protein oxidation; as a marker of oxidative damage to proteins, carbonyls have been confirmed to accumulate during aging (age-related disease) and inflammation [6,7]. A slight modification of the previously described methods was used in this experiment [8,9]. Membrane and cytosol extract samples were analyzed. Both fractions were incubated in 10 mM 2,4-dinitrophenylhydrazine (DNPH) prepared in 2 N HCl for 1 hr in the dark. Next, 20 μL of 20% TCA was added to the tubes, and the mixture was incubated at room temperature for 10 min. The tubes were then centrifuged at 12000 x g for 3 min to obtain the protein pellet. Finally, the precipitates were resuspended in 100 μL of 1 N NaOH and incubated for 15 minutes at 37ºC. The carbonyl content was determined in the centrifuged product by the absorbance at 360 nm (VERSAmax Molecular Devices, Sunnyvale, CA, USA). The carbonyl content was calculated using the Lambert-Beer equation ($A= e \times C \times b$), where the absorption coefficient (e) is 22/m/cm, and the results were expressed as nmol/mg protein.

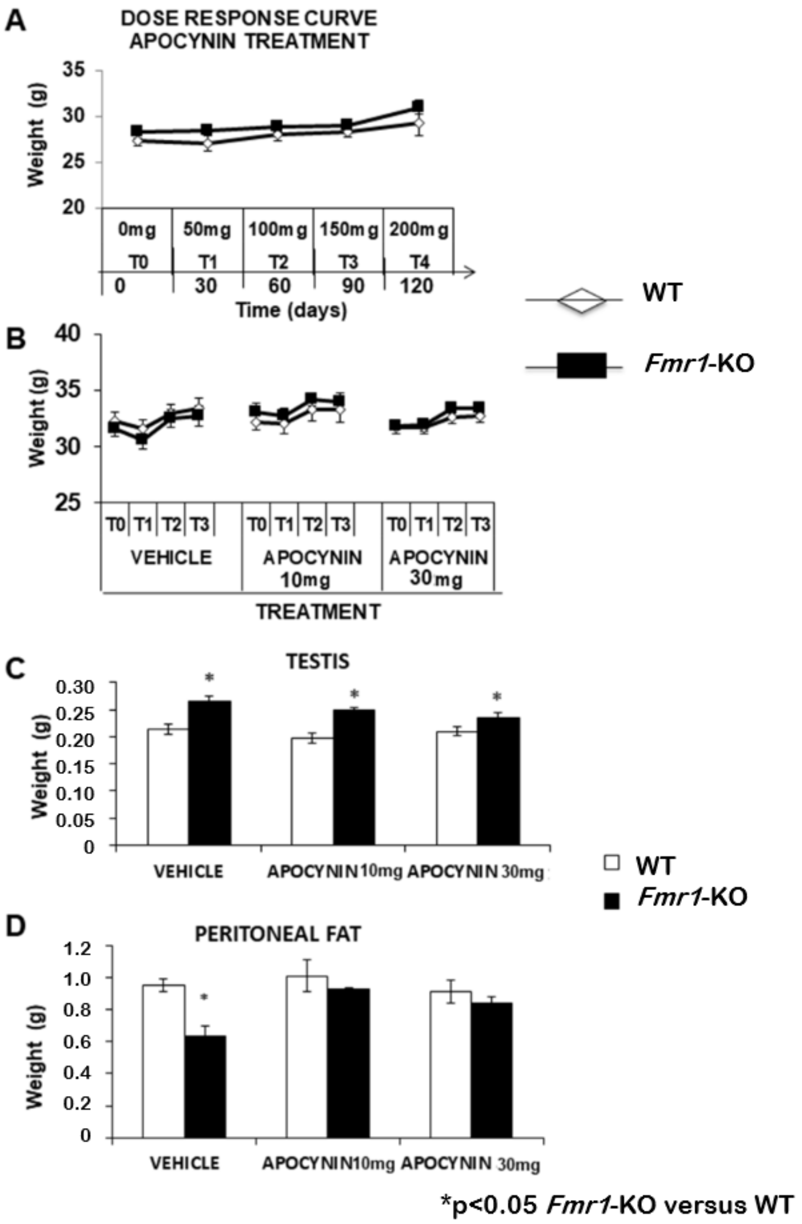


**Figure S1:** Measure of Apocynin tolerance in mice (**A**). Evaluation of the effects of incremental doses (50 mg per 30 days of the experiment) on weight over 120 days. (**B**). Evaluation of the effect on weight of the application of vehicle, 10 mg/kg/day Apocynin and 30 mg/kg/day Apocynin over 30 days. Apocynin does not significantly alter the weight of mice. The data shown represent the mean weight per group of 6 animals ± SEM (*p <0.05 WT-controls vs Fmr1-KO). Testis (**C**) and peritoneal fat (**D**) weight after treatment with vehicle and two doses of Apocynin (10 mg/kg/day and 30 mg/kg/day). In the testicles, there was a trend toward a reduction of weight between the WT-controls groups and Fmr1-KO group, but macroorchidism was maintained in response to these Apocynin doses. However, the differences in peritoneal fat weight were normalized after chronic treatment with Apocynin. The data shown represent the average weight of the tissue in groups of 6 animals ± SEM.

**Supplementary References**


1. Crusio, W.; Schwegler, H. Hippocampal Mossy Fiber Distribution Covaries with Open-Field Habituation in the Mouse. *Behavioural Brain Research* 1987, *26*, 153–158, doi:10.1016/0166-4328(87)90163-X.
2. Leger, M.; Quiedeville, A.; Bouet, V.; Haelewyn, B.; Boulouard, M.; Schumann-Bard, P.; Freret, T. Object Recognition Test in Mice. *Nat Protoc* 2013, *8*, 2531–2537, doi:10.1038/nprot.2013.155.

3. Lister, R.G. The Use of a Plus-Maze to Measure Anxiety in the Mouse. *Psychopharmacology (Berl)* 1987, *92*, 180–185, doi:10.1007/BF00177912.
4. Phillips, R.G.; LeDoux, J.E. Differential Contribution of Amygdala and Hippocampus to Cued and Contextual Fear Conditioning. *Behavioral Neuroscience* 1992, *106*, 274–285, doi:10.1037/0735-7044.106.2.274.
5. Ohkawa, H.; Ohishi, N.; Yagi, K. Assay for Lipid Peroxides in Animal Tissues by Thiobarbituric Acid Reaction. *Anal Biochem* 1979, *95*, 351–358, doi:10.1016/0003-2697(79)90738-3.
6. Dalle-Donne, I.; Giustarini, D.; Colombo, R.; Rossi, R.; Milzani, A. Protein Carbonylation in Human Diseases. *Trends Mol Med* 2003, *9*, 169–176, doi:10.1016/S1471-4914(03)00031-5.
7. Dean, R.T.; Fu, S.; Stocker, R.; Davies, M.J. Biochemistry and Pathology of Radical-Mediated Protein Oxidation. *Biochemical Journal* 1997, *324*, 1–18, doi:10.1042/bj3240001.
8. Carty, J.L.; Bevan, R.; Waller, H.; Mistry, N.; Cooke, M.; Lunec, J.; Griffiths, H.R. The Effects of Vitamin C Supplementation on Protein Oxidation in Healthy Volunteers. *Biochem Biophys Res Commun* 2000, *273*, 729–735, doi:10.1006/bbrc.2000.3014.
9. Levine, R.L.; Garland, D.; Oliver, C.N.; Amici, A.; Climent, I.; Lenz, A.-G.; Ahn, B.-W.; Shaltiel, S.; Stadtman, E.R. [49] Determination of Carbonyl Content in Oxidatively Modified Proteins. In *Oxygen Radicals in Biological Systems Part B: Oxygen Radicals and Antioxidants*; Methods in Enzymology; Academic Press, 1990; Vol. 186, pp. 464–478.
